# Therapeutic potential of plant-derived exosome-like nanoparticles for CNS diseases: advancements in preparation and characterization

**DOI:** 10.1080/07853890.2025.2537345

**Published:** 2025-07-28

**Authors:** Mengmeng Liu, Hongli Wang, Yizhuang Yang, Jianhua Wang, Zhen Sun, Shiyuan Zhao, Weihua Kong, Wenxue Sun, Cuiyun Wang, Lei Feng

**Affiliations:** a Department of Pharmacy, Jining No. 1 People’s Hospital, Shandong First Medical University, Jining, China; b Department of Pharmacy, Guilin Medical University, Guilin, China; c Shandong University of Traditional Chinese Medicine, Jinan, China; d Translational Pharmaceutical Laboratory, Jining No. 1 People’s Hospital, Shandong First Medical University, Jining, China; e Institute of Translational Pharmacy, Jining Medical Research Academy, Jining, China; f Department of Neurosurgery, Jining No.1 People’s Hospital, Shandong First Medical University, Jining, China

**Keywords:** Exosomes, plant-derived exosome-like nanoparticles, CNS diseases, drug delivery systems

## Abstract

**Background:** Central nervous system (CNS) diseases pose significant therapeutic challenges owing to their complex pathophysiology and the restrictive nature of the blood-brain barrier (BBB).

**Introduction:** Plant-derived exosome-like nanoparticles (PDELNs) exhibit intrinsic bioactivity, low immunogenicity, and ability to cross the BBB, making them a promising therapeutic strategy for CNS disorders.

**Discussion:** This review explores the preparation, isolation, and characterization of PDELNs, with a focus on their therapeutic applications in CNS diseases. We also discuss different administration routes and the impact of functional modifications on BBB penetration.

**Conclusions: **PDELNs offer scalability, biocompatibility, and natural pharmacological activity, which make them attractive candidates for CNS drug delivery. However, challenges remain, including standardized isolation, elucidation of BBB penetration mechanisms, and long-term stability and safety. Future research should focus on clarifying their mechanisms of action, optimizing preparation methods, and validating their long-term safety to facilitate the clinical translation of PDELNs.

**KEY MESSAGES**
- PDELNs are derived from abundant plant sources, exhibit favorable physicochemical properties.
- PDELNs offer new possibilities for CNS therapies owing to their low immunogenicity, high biocompatibility, and ability to cross the BBB.

## 1. Introduction

Central nervous system (CNS) diseases are a complex and diverse class of neurological disorders, including neurodegenerative, autoimmune, cerebrovascular, and traumatic conditions, such as Alzheimer’s disease (AD), Parkinson’s disease (PD), multiple sclerosis, and stroke. Approximately one-sixth of the global population is affected by CNS diseases [1]. Despite some progress in disease management in recent years, existing treatments have focused primarily on alleviating symptoms rather than providing a cure. This limitation is mainly due to the blood-brain barrier (BBB). The BBB, with its tightly structured composition, prevents most therapeutic molecules from entering the brain, significantly limiting the effectiveness of drug delivery [2]. Therefore, developing drug delivery systems that can effectively cross the BBB has become a critical challenge for the treatment of CNS diseases.

Several drug delivery strategies have been developed to overcome the BBB, including monoclonal antibodies (mAbs), small-molecule drugs, lipid nanoparticles (LNPs), and viral vectors [3]. However, this approach also has several limitations. Despite their specificity, mAbs are large biomolecules with poor BBB penetration and require invasive delivery methods or chemical modifications to enhance transcytosis [4]. Small-molecule drugs cross the BBB more efficiently but often suffer from low selectivity, rapid clearance, and potential off-target effects, which limit their long-term efficacy [5]. LNPs and viral vectors have shown promise for brain-targeted drug delivery; however, toxicity, immunogenicity, and long-term stability remain major concerns [5,6]. These limitations highlight the urgent need for novel, biocompatible, and scalable drug delivery systems for treating CNS diseases.

In recent years, extracellular vesicles (EVs) have shown great potential as bioactive carriers for drug delivery and intercellular communications. EVs are released by various types of cells under different physiological conditions and are primarily categorized into ectosomes and exosomes. Exosomes typically range from 40 to 160 nm in diameter and originate from the endosomal pathway, which can carry diverse biomolecules, such as DNA, RNA, lipids, and proteins [7]. Owing to their biocompatibility, tissue-targeting ability, and drug-loading capacity, mammalian exosomes have emerged as potential candidate carriers for delivering CNS disease drugs [8]. In contrast to the extensive research on mammalian exosomes, studies on plant-derived extracellular vesicles (PDEVs) have begun relatively recently. As early as the 1960s, Halperin et al. observed exosome-like structures in plant cells using transmission electron microscopy (TEM) for the first time [9]. It was not until 2009 that de la Canal et al. isolated vesicles containing exosome marker proteins from the apoplast of sunflowers and analyzed their morphology and composition[10]. Subsequent studies have shown that various plants, including *Arabidopsis thaliana*, tobacco, and mistletoe, can release exosomes[11]. In 2013, Zhang et al. isolated exosome-like nanoparticles (ELNs) from grape juice. They discovered that these nanoparticles could target intestinal stem cells, promote tissue repair, and effectively protect mice from dextran sulfate sodium-induced colitis [12]. This has sparked growing interest in the study of plant-derived exosome-like nanoparticles (PDELNs).

PDELNs share some functions and components similar to mammalian exosomes but differ in their biogenesis pathways. As the biogenesis pathway of PDELNs is yet to be fully understood, they are not entirely synonymous with PDEVs. This uncertainty led to conceptual and practical challenges in this investigation. However, PDELNs are more manageable to isolate in large quantities and yield higher than PDEVs, making PDELNs research the main direction for studying in biomedical applications [13]. The potential of PDELNs in the treatment of CNS diseases is gradually emerging. Some studies have suggested that PDELNs can cross the BBB and deliver therapeutic cargo to damaged neural tissues through non-cell-autonomous mechanisms [14]. Owing to their wide availability, low immunogenicity, and ease of production, PDELNs show great potential as drug delivery carriers. However, several challenges remain in their application to CNS diseases, including optimizing drug-loading capacity, enhancing the efficiency of BBB crossing, and ensuring long-term stability in different disease models [15,16]. This review aims to explore the potential applications of PDELNs in treating CNS diseases; analyze related studies on their preparation, characterization, and *in vivo* and *in vitro* evaluation; and propose future challenges and development directions in the field of neurology.

## 2. Preparations of PDELNs

**Figure 1.**
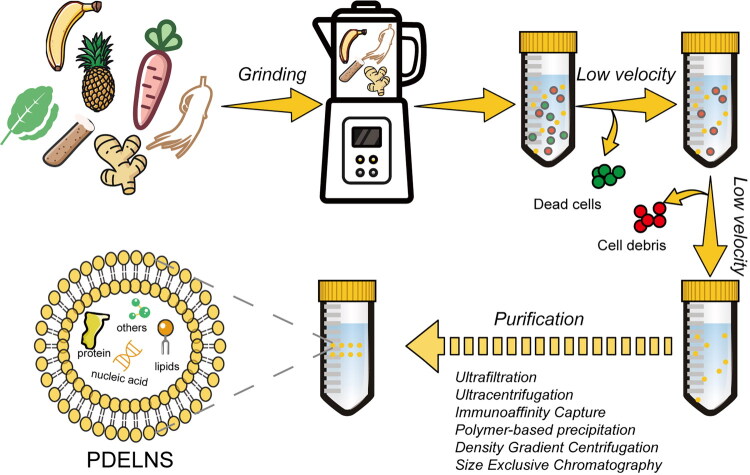
Illustration of the grinding, centrifugation, and partial purification steps in the preparation of PDELNs.

According to the recommendations of the International Society for Extracellular Vesicles (ISEV) in their 2018 MISEV guidelines, the goals for the isolation of EVs can be categorized into four types: high yield with low specificity, moderate yield with moderate specificity, low yield with high specificity, and high yield with high specificity [17]. To date, no method has been able to balance yield and specificity. Therefore, when preparing PDELNs, as shown in Figure 1, the selection of these steps should be adjusted based on specific research needs. Researchers should choose appropriate isolation methods rather than simply seeking purity.

### 2.1. Plant tissue pretreatment

Before isolating PDELNs, pretreatment of plant tissues is crucial in their preparation. Different pretreatment methods directly affect the purity and yield of the final nanoparticles. Two standard methods were used: tissue disruption and infiltration centrifugation.

#### 2.1.1. Tissue disruption method

Tissue disruption mechanically breaks down plant tissues, producing plant sap containing PDELNs and cell debris. This was followed by low-speed centrifugation to remove the large particles and cell debris. After multiple rounds of low-speed and high-speed centrifugation, the PDELNs can be enriched. This method is advantageous for processing large amounts of material and is suitable for preparing high-yield PDELNs [18]. However, because plant cells are destroyed during the disruption process, organelle membranes and cell debris may mix, potentially affecting the purity of the PDELNs. Recent studies have detected proteins from the cytoplasm, nucleus, and organelles in leaf nanoparticle protein analyses, indicating that the tissue disruption method may introduce endogenous contaminants [19]. Despite this, because of the simplicity, efficiency, and high concentration of PDELNs obtained, the tissue disruption method remains the most widely used preparation technique. It is particularly suitable for research requiring higher yields and relatively lower purities.

#### 2.1.2. Tissue infiltration centrifugation method

Tissue infiltration centrifugation was initially used to extract intercellular fluid from plant cells. This process involves injecting a specific isolation buffer into the plant tissues and using a vacuum device to infiltrate the tissue. After infiltration, low-speed centrifugation collects intercellular fluid from plant cells. Compared with the tissue disruption method, this approach better preserves the integrity of cell structures, resulting in PDELNs with higher purity, closely resembling true exosomes. However, this method produces a lower amount of PDELNs and may be affected by cell damage and dilution of intercellular fluid [20]. Additionally, this method is suitable for leaf tissues and has limited applications in roots, stems, and fruits.

### 2.2. Isolation and purification methods for PDELNs

After the pretreatment of plant tissues, the isolation and purification of PDELNs are key steps to ensure high-quality samples. Current methods include ultracentrifugation, density gradient centrifugation, ultrafiltration, immunoaffinity capture, and size-exclusion chromatography. Each method has distinct advantages and limitations in terms of purity and yield.

To provide a clearer comparison, Table 1 summarizes the key characteristics of the different PDELNs isolation techniques, including their advantages and limitations.

**Table 1.** Methods for purification and isolation of PDELNs.

Method	Purity	Yield	Advantages	Limitations
Ultracentrifugation	High	Moderate	High purity, efficient removal of contaminants	Time-consuming, requires expensive equipment, may cause vesicle deformation
Density Gradient Centrifugation	Very high	Low	High separation efficiency, prevents vesicle aggregation	Time-consuming, potential contamination from gradient media, not suitable for large-scale production
Ultrafiltration	Moderate	High	Fast, simple, maintains vesicle integrity	Requires membrane optimization, risk of clogging or vesicle loss
Size-Exclusion Chromatography (SEC)	Very high	Low	High purity, removes free proteins and small contaminants	Low throughput, requires multiple processing steps
Immunoaffinity Capture	Very high	Low	Highly specific, isolates targeted PDELN subtypes	Requires specific markers, costly antibodies or magnetic beads
Polymer-Based Precipitation (e.g. PEG)	Moderate	Very high	Cost-effective, high recovery, scalable	Co-precipitation of contaminants, requires additional purification

#### 2.2.1. Ultracentrifugation and density gradient centrifugation

Ultracentrifugation is the most widely used separation method based on differences in the sedimentation coefficients of particles under high-speed centrifugation. Particles with varying sedimentation coefficients can be separated under different centrifugation conditions. Typically, large particles and cell debris are first removed by multiple rounds of low-speed centrifugation, followed by ultracentrifugation at forces of up to 40,000 × *g* or higher to enrich PDELNs. For example, Huang et al. used different centrifugal forces to obtain three distinct subtypes of PDELNs from the apoplastic washing fluid (AWF) of *Arabidopsis thaliana* leaves [20]. However, ultracentrifugation has certain drawbacks, particularly under high centrifugal forces that can cause particle deformation. To prevent deformation or damage to the PDELNs under high forces, an appropriate concentration of sucrose or iodixanol can be added at the bottom of the centrifuge tube as an isotonic buffer. Ultracentrifugation is often combined with density gradient centrifugation to separate nanoparticles of different sizes and densities. Sucrose and iodixanol are the two most widely used density-gradient media in PDELNs research. Zhuang et al. used discontinuous sucrose gradients for ultracentrifugation, effectively removing unbound substances and impurities and purifying grapefruit-derived exosome-like nanoparticles (GDELNs) [21]. Seo et al. used sucrose gradient separation to extract nanoparticles from ginseng, demonstrating their significant effects on inhibiting osteoclast differentiation [22]. Rutter and Innes used MES buffer (pH 6), which mimics the natural environment of *Arabidopsis rosette* apoplastic fluid, to treat AWF, followed by purification of vesicles using a discontinuous gradient based on iodixanol (OptiPrep gradient) [23]. Density gradient centrifugation offers the advantages of a high separation efficiency and low particle deformation rates. However, density media such as sucrose may inevitably be incorporated into the particles, affecting their interaction with specific molecules and influencing subsequent experiments [24]. Given the potential impact of density gradient media on PDELNs components and the time-consuming nature of the process, density gradient centrifugation may not be suitable for the large-scale production of PDELNs.

#### 2.2.2. Ultrafiltration

Ultrafiltration (UF), a size-based separation technique, has been widely applied to isolate PDELNs. Nanoporous membranes are used to retain larger particles under fluid pressure or low-speed centrifugation, allowing smaller vesicles to pass through. The two common operation modes for ultrafiltration are pressure-driven and centrifugal ultrafiltration, which are often considered to provide better separation efficiency. For example, Kang et al. successfully isolated high concentrations of onion-derived ELNs using a centrifugal filtration device [25].

In practice, the selection of the centrifugation force and time is critical, as excessive force or extended centrifugation may damage the ultrafiltration membrane, thereby affecting the separation efficiency. Maintaining moderate operating conditions ensures the successful separation of the ELNs. Ultrafiltration is often combined with size-exclusion chromatography (SEC) to enhance the purity of separation. For instance, Kim and Rhee successfully purified high concentrations of carrot-derived exosome-like nanoparticles (Carex) using a combination of ultrafiltration and SEC [26]. Although ultrafiltration offers the advantages of speed, simplicity, and efficiency, removing large membrane structures from the sample before processing to prevent their breakdown into smaller vesicles could affect the purity of the subsequent analyses. In general, as an emerging technique for isolating PDELNs, ultrafiltration offers significant advantages in maintaining bioactivity and ease of operation.

#### 2.2.3. Size-exclusion chromatography

SEC separates particles based on molecular size differences and is another commonly used method for purifying PDELNs. The advantage of SEC lies in its ability to effectively remove free proteins and other small-molecule contaminants, resulting in highly pure and size-uniform PDELNs. This method is often combined with other separation techniques to improve the efficiency. You et al. compared ultracentrifugation, polyethylene glycol (PEG) precipitation, and SEC combined with ultrafiltration to isolate exosome-like nanovesicles from cabbage. They found that vesicles isolated using SEC showed relatively uniform peaks and high purities [27]. Additionally, Berger et al. used SEC to separate orange juice-derived nanovesicles obtained by ultracentrifugation and successfully isolated small and large vesicles [28].

#### 2.2.4. Immunoaffinity capture

Immunoaffinity capture is a technique that uses exosomal surface markers to purify specific vesicle subpopulations. In mammalian exosomes, antibodies or peptides are typically used to capture vesicles based on specific lipids, polysaccharides, or proteins on their surfaces. This technique has been widely applied to isolate mammalian exosomes using common markers such as CD9, CD63, and CD81. However, research on the specific markers of PDELNs is limited. He et al. successfully isolated TET8-positive EVs from *Arabidopsis* using a combination of sucrose density gradient centrifugation and immunoaffinity capture techniques [29]. Although there are no universally applicable markers for PDELNs, immunoaffinity capture remains the most precise method for isolating specific subtypes of EVs. Immunoaffinity capture can effectively prevent the co-isolation of non-vesicular cytoplasmic contaminants and other unwanted vesicles through the interaction between antigens and antibodies. In the future, it will be necessary to develop more marker-specific antibodies and magnetic beads, and optimize methods to efficiently capture and release PDELNs from magnetic beads to facilitate their use in subsequent experiments.

#### 2.2.5. Polymer-based precipitation method

The polymer-based precipitation method utilizes superhydrophilic polymers for coprecipitation. The first precipitation medium was PEG, which can bind hydrophobic proteins and lipid molecules for co-precipitation. Kalarikkal et al. developed a ginger-derived exosome-like nanoparticles (G-ELNs) purification method based on PEG-6000, demonstrating an efficiency comparable to ultracentrifugation while offering a cost-effective alternative [30]. Nguyen et al. incubated nanovesicles from *Brassica oleracea* buds with different concentrations of PEG, ultimately isolating PEG-coated PDELNs. These nanovesicles exhibit high stability without loss of bioactivity [31].

Commercial kits based on polymer precipitation are widely used to isolate plant-derived exosomes. Jang et al. evaluated the effectiveness of ultracentrifugation, the ExoQuick system (a polymer-based exosome precipitation method), and a combination of both in isolating exosomes from ginseng. They found that the purity of exosomes isolated using the ExoQuick method was higher than that obtained with ultracentrifugation alone, and combining the two methods significantly improved isolation purity, achieving more than double the purity compared to that obtained with ultracentrifugation alone. Moreover, this combined method is suitable for isolating stable, high-purity exosomes from *Arabidopsis* [32].

## 3. Physical characteristics of PDELNs

Once PDELNs have been successfully isolated and purified, it is essential to characterize their physical properties to confirm their structural and functional attributes. The evaluation focused mainly on morphology, zeta potential, and particle size distribution.

### 3.1. Morphology

The morphological analysis of PDELNs is a critical step in their physical characterization. Several microscopy techniques can be used to observe and identify their morphology, including TEM, atomic force microscopy (AFM), scanning electron microscopy (SEM), and cryo-electron microscopy (Cryo-EM). Among these, TEM is an important technique for assessing the ultrastructure of nanoparticle vesicles and is the most used method for obtaining nanometer-scale resolution images of PDELNs. Lange et al. isolated EVs from the extracellular fluid of sunflower seedlings and characterized them using TEM [33]. Huang et al. used TEM to observe vesicles in P100 fractions prepared from AWF extracted by different methods, assessing which method reduced impurity contamination during exosome preparation [20]. Although TEM provides high-resolution images, the samples must be fixed and dehydrated before measurement, which can cause PDELNs to appear in an artificial cup-shaped form, not reflecting their actual morphological characteristics.

To observe particles in a more natural state, Cryo-EM offers direct imaging of particles in near-native conditions, reliably providing information about the size and shape of EVs. Jang et al. initially observed ELNs using TEM, but the technique did not clearly distinguish whether they were exosomes. They then performed further analysis using cryo-EM, which confirmed the presence of ginseng-derived exosome-like nanoparticles (GENs) in the sample [32]. Similarly, Garaeva et al. used Cryo-EM to identify grapefruit-derived particles, finding that these particles mainly exhibited a round vesicular shape composed of a lipid bilayer with an average thickness of 5.3 ± 0.8 nm, defining them as GDELNs [34].

Compared to the limited understanding of PDELNs, which were thought to exist only in seed and leaf debris, Chukhchin et al. reported the use of SEM to observe EVs extracted from branch and stem samples of angiosperms and gymnosperms. SEM revealed the characteristic cup-shaped structure of exosomes in their dried state [14]. In addition to electron microscopy-based methods, AFM is a high-resolution scanning probe microscope that offers unique advantages for morphological analysis. AFM not only provides the surface topography of the particles but also delivers mechanical information such as stiffness and adhesion properties. Sharma et al. used AFM imaging to analyze avocado-derived ELNs and found that their size was consistent with the results obtained by TEM analysis [35]. Garaeva et al. performed morphological identification of GDELNs using Cryo-EM and conducted a detailed analysis of their surface topography using AFM [34]. However, AFM is less frequently applied to PDELNs than electron microscopy, probably because researchers are generally more focused on their morphological and structural characteristics than on their mechanical properties such as stiffness and adhesion.

### 3.2. Particle size and zeta potential

Dynamic light scattering (DLS) and nanoparticle tracking analysis (NTA) are two standard methods for measuring the particle size and zeta potential. DLS converts the diffusion coefficient of particles into a hydrodynamic diameter distribution, allowing for quick assessment of the particle size and zeta potential of PDELNs. It has advantages, such as a small sample volume and high sensitivity. However, DLS has a relatively low resolution and struggles to distinguish between particles with small size differences [36]. In contrast, NTA is a high-throughput single-particle analysis method that tracks the Brownian motion of particles to determine their average velocity and diffusion rate, thereby quantitatively assessing particle size distribution and concentration. NTA offers higher resolution and good repeatability, and is less affected by the scattering intensity of large particles. However, NTA requires specific particle trajectory lengths and sample concentrations to ensure accuracy because the samples must fall within a certain concentration range [37].

PDELNs from different sources or isolated using other methods may exhibit variations in particle size and zeta potential, with zeta potential typically showing negative values. When DLS and NTA are combined, a more comprehensive evaluation of the physical properties of the PDELNs can be achieved. DLS is suitable for quickly assessing the overall size distribution, whereas NTA provides more detailed data on size distribution and concentration. For example, Garaeva et al. used DLS and NTA to characterize GDELNs. NTA provided data on vesicle size distribution and concentration, whereas DLS supplemented size measurements revealed a multimodal distribution of particles [34].

The physicochemical properties of PDELNs play a crucial role in their bioactivity and interactions with cells, thereby directly influencing their uptake efficiency and stability. Particle size is a key determinant of the ability of nanovesicles to traverse biological barriers and interact with the target cells. Studies have suggested that nanovesicles smaller than 200 nm are more likely to enter cells *via* clathrin-mediated endocytosis [38], whereas larger vesicles are more prone to recognition and rapid clearance by the mononuclear phagocyte system, which affects their *in vivo* stability and functionality [39]. Additionally, zeta potential is a critical parameter for evaluating the colloidal stability of nanoparticles [40]. Generally,the zeta potential of exosomes ranges from −6 to −30 mV, with values below −20 mV indicating high colloidal stability [41,42]. Most PDELNs exhibited zeta potentials within this range [43], which helped maintain their stability in suspension and minimized aggregation.

## 4. Biochemical characteristics of PDELNs

Many studies have shown that PDELNs comprise various biomolecules, including lipids, proteins, and RNA, demonstrating their potential biological functions and broad application prospects. PDELNs are widely found in different plants and their composition and function depend on the type of source cells. To gain a deeper understanding of the biological characteristics and application potential of PDELNs, a comprehensive analysis and characterization of their chemical composition is crucial [44]. With advances in technology, multiple analytical methods can be used to precisely characterize the composition of PDELNs. Commonly used techniques include enzyme-linked immunosorbent assays, sodium dodecyl sulfate-polyacrylamide gel electrophoresis [45], liquid chromatography-tandem mass spectrometry [46,47], small RNA sequencing, and fluorescence analysis [48]. These methods allow the in-depth analysis of lipids, proteins, and RNA in PDELNs. Colorimetric methods, fluorescence detection, and Raman spectroscopy have also been used to detect other biomolecules [17,49]. These techniques can fully reveal the chemical structures and potential biological functions of PDELNs.

### 4.1. Lipids

Lipids are a significant component of PDELNs that form the fundamental structure of vesicle membranes [50]. Analyses have shown that PDELNs are rich in phospholipids and glycerolipids, which play key roles in vesicle stability, cell uptake, and biological functions [12]. Phosphatidic acid is an important lipid that regulates membrane fusion and fission and promotes vesicle uptake by rearranging the cytoskeleton [51]. Additionally, PDELNs contain phosphatidylethanolamine, phosphatidylcholine, phosphatidylinositol, and diacylglycerols. Cholesterol has not been detected in PDELNs, distinguishing them from EVs in animal cells [52].

### 4.2. Proteins

The functional protein components in PDELNs deserve in-depth investigation, particularly proteins that are enriched or deficient in PDELNs [13]. Studies have shown that vesicles from plants, such as ginger, ginseng, and lemon, contain abundant cytoplasmic proteins [53–55]. Additionally, small amounts of membrane transport proteins such as aquaporins have been identified in PDELNs, further indicating the complex biological functions of these vesicles [56].

Beyond studying proteins that are enriched or lacking in PDELNs, identifying protein markers is critical for understanding the origin and biological function of these vesicles. Protein markers are commonly found in most EVs and provide important information about vesicle origin, intracellular localization, and secretion mechanisms. For example, in mammalian exosomes, the transmembrane proteins CD9, CD81, and CD63 are typical markers [57]. However, research on specific markers for PDELNs is limited, and to date, only a few candidate markers have been identified.

In *Arabidopsis*, penetration protein 1 (PEN1) is highly enriched within these vesicles, and is likely closely related to the structure of the extracellular membrane [18]. TET8 protein has also been found on the membrane surface of *Arabidopsis*-derived ELNs [58]. However, further experimental validation is required to determine whether PEN1 or TET8 can serve as universal markers for PDELNs from different plants.

### 4.3. Rna

PDELNs can carry various RNA molecules including mRNA and miRNAs. Some studies suggest that PDELNs contain hundreds of miRNAs, which can be transferred between cells through PDELNs, regulating gene expression and protein synthesis in recipient cells and affecting their functions [59,60]. For example, rice-derived miR-168a can enter the circulatory system in mice through the gastrointestinal tract and regulate the expression of LDLRAP1, thereby reducing the clearance of low-density lipoprotein in mouse plasma [61]. PDELNs may play a potential biological role in intercellular communication and gene regulation.

Different types of small RNA molecules have been identified in the PDELNs. Baldrich et al. discovered that PDELNs from *Arabidopsis thaliana* contain a type of short RNA, known as tyRNA, with lengths ranging from 10 to 17 nucleotides. These short RNAs may be involved in long-distance transport and gene regulation in target cells, thereby expanding the role of PDELNs in gene regulation. As the functions of these short RNAs are still being explored, further research is needed to understand their possible regulatory mechanisms and biological effects [62,63]. Moreover, studies have shown that the RNA composition of PDELNs may vary significantly among plant species [64], which could be related to the physiological characteristics of the plant, RNA biosynthesis pathways, and their functions. Therefore, investigating the species-specific RNA composition of PDELNs will help to reveal their unique biological roles and potential mechanisms in cross-species communication and gene regulation.

### 4.4. Metabolites

In addition to lipids, proteins, and RNA, PDELNs may contain various metabolites. These metabolites have potential biological effects on the bioactivity of the PDELNs. For instance, studies have shown that ELNs are rich in pectic polysaccharides, specifically galacturonic acid. This polysaccharide enhances the tolerance of *Lactobacillus rhamnosus* GG (LGG) to bile by regulating the degradation of specific tRNAs in LGG [65]. As research on PDELNs has advanced, these metabolites may present broad prospects for future therapeutic applications.

## 5. Advantages and limitations of PDELNs

### 5.1. High yield

PDELNs demonstrate significant advantages in terms of yield, which is especially important in biotechnology and medical research. Although most studies are experimental, the high yield and diverse plant sources of PDELNs offer great potential for future applications. The large-scale production of PDELNs can be achieved using plant cell culture technology. For example, Ou et al. established a plant cell culture system using *Catharanthus roseus*, successfully obtaining gram-scale nanovesicles from the culture medium with similar physical characteristics and biological activities, producing three times more nanovesicles than traditional methods [66]. Additionally, the short extraction cycle of PDELNs enables rapid acquisition of large quantities of products, effectively meeting the demands of practical applications. Given the diversity of plant species, almost all plants can serve as potential sources of PDELNs, providing ample and varied options for raw materials [11].

### 5.2. Stability

The stability of PDELNs is crucial for maintaining their biological functions; however, they still face several challenges. Various factors, including storage conditions, pH, ultrasonication, and temperature, can affect the stability of PDELNs. Some studies have shown that yam-derived ELNs retain their bioactivity after storage at −80 °C for one year, indicating good compositional stability [67]. Additionally, Ou et al. evaluated the stability of *Catharanthus roseus* (L.) Don leaves-derived exosome-like nanovesicles (CLDENs) using several methods. The results demonstrated that CLDENs could remain stable in extreme pH environments, resist degradation by DNase, RNase, and protease, and maintain high stability in simulated gastrointestinal fluids [66].

To further assess the stability of PDELNs under physiological conditions, Zhu et al. cultured Portulaca oleracea L-derived ELNs in a simulated gastrointestinal environment. The results showed that these nanoparticles could pass through the acidic environment of the stomach, reach the small intestine, and be absorbed, demonstrating excellent stability [68]. Similarly, Umezu et al. compared the degradation of exogenous miRNAs and Acerola ELN-miRNA complexes in acidic, alkaline, and RNase environments. Their results showed that miRNAs within Acerola ELN-miRNA complexes were not fully degraded under any of these conditions [69]. However, a recent study found that ultrasonication significantly reduced the ability of GENs to regulate macrophage polarization, indicating that the intact structure of PDELNs is critical for their biological function. Similarly, another study pointed out that pretreatment with the chemical detergent Triton X-100 resulted in a significant reduction in PDELNs activity [70]. Therefore, further research is needed to investigate the stability of different PDELNs to ensure consistent physicochemical properties while maintaining their bioactivity.

### 5.3. Toxicity and immunogenicity of PDELNs

PDELNs are primarily derived from edible plants and exhibit significantly reduced toxicity to the human body compared to artificial nanoparticles and EVs secreted by mammalian cells. Studies have shown that PDELNs exhibit good safety profiles both *in vitro and in vivo*. For instance, Chen et al. conducted experiments using ELNs extracted from cucumber pulp, showing that these nanovesicles did not cause hemolysis when injected intravenously into mice, and the levels of pro-inflammatory cytokines, such as tumor necrosis factor-α (TNF-α) and interleukin-1β (IL-1β), did not increase significantly. These results suggest that PDELNs elicit a mild biological response *in vivo* without triggering a significant inflammatory reaction. Furthermore, histological examination revealed no apparent damage to major organs, such as the heart, liver, spleen, and kidneys in mice treated with PDELNs, demonstrating their non-toxicity and safety [71]. This finding is significant for the potential application of PDELNs in clinical therapies, particularly in drug delivery systems.

Wang et al. demonstrated that ginger-derived exosome-like nanoparticles (GExos) did not cross the placental barrier in pregnant mice, indicating their safety for fetuses [54]. Given the safety concerns associated with drug delivery during pregnancy, this characteristic of PDELNs positions them as potential preferred carriers for drug delivery during pregnancy. Although current studies suggest that PDELNs exhibit low toxicity and immunogenicity, further research is needed to systematically evaluate their safety across different routes of administration. Moreover, factors such as the plant source, extraction and processing methods, and specific composition of PDELNs could influence their biocompatibility and safety. Therefore, in-depth studies on the long-term safety and possible adverse effects of PDELNs are a priority for future research.

### 5.4. Biological distribution

Several studies have focused on the internalization mechanisms and biodistribution of PDELNs *in vivo*. Research has shown that PDELNs can be taken up by various mammalian cells, including immune and non-immune cell types, and show wide adaptability under normal and pathological conditions [54,72]. In these cells, phagocytosis and endocytosis are the primary mechanisms of PDELNs uptake [13]. Further studies have revealed that the specific lipid composition of PDELNs plays a crucial role in regulating cellular uptake [73]. Understanding the mechanisms of PDELNs uptake across different organ systems is essential for elucidating their biological functions and optimizing their applications in biotherapy and drug delivery.

The biodistribution of PDELNs is closely related to their route of administration. Oral administration is the most common method of administering PDELNs, with most vesicles accumulating in the gastrointestinal tract [74]. This method is considered to be highly safe because of the well-established safety of edible plants. In contrast, intravenous and intraperitoneal injections lead to PDELNs, which are predominantly distributed in the liver and spleen. In one study, DiR-labeled GENs were injected intravenously and intraperitoneally into C57BL/6 mice. The results showed that most PDELNs accumulated in the liver and spleen within 72 h [75]. This difference highlights the importance of administration routes for optimizing the therapeutic effects of PDELNs, especially for targeting specific organs or tissues.

Xu et al. observed fluorescent solid signals in the brain after feeding C57BL/6 mice DiR-labeled PDELNs, suggesting that PDELNs may potentially cross the BBB [76]. In the same mouse model, researchers designed GDELNs coated with folic acid (FA) and polyethyleneimine to target GL26 brain tumors. These PDELNs successfully delivered miR-17 through intranasal administration, effectively delaying tumor growth [77]. In addition to oral, intravenous, and intraperitoneal administration, PDELNs also serve as efficient carriers for transdermal drug delivery [78]. For example, in a full-thickness skin defect model, the combination of chemokine-crosslinked hydrogels with GENs effectively transferred miRNA, significantly promoting neurodifferentiation of bone marrow mesenchymal stem cells (BMSCs) [55]. Furthermore, Yepes-Molina et al. found that broccoli-derived ELNs could be successfully transported to keratinocytes, where plant proteins are detected within the cytoplasmic membrane of keratinocytes [68].

Understanding the *in vivo* biodistribution of PDELNs is crucial for developing targeted therapies for specific diseases. The biodistribution characteristics of PDELNs may directly influence their therapeutic efficacy in various scenarios.

### 5.5. Limitations of PDELNs

Despite the promising potential of PDELNs, several challenges remain in their therapeutic application. Two major concerns are the variability of plant sources and the need for batch-to-batch consistency, both of which are crucial for reproducibility.

The composition and therapeutic effects of PDELNs depend on plant species, growth conditions, and extraction methods. ELNs from different plants vary in lipid composition, protein cargo, and bioactive molecules, leading to differences in their biological activity [79,80]. For example, GExos exhibited strong anti-inflammatory effects, whereas GDELNs exhibited enhanced drug delivery efficiency because of their lipid profile [21,55].

The size, morphology, and stability of ELNs vary not only between different plant species but also among different parts of the same plant [66]. Jang et al. investigated the effects of three extraction methods and found that they significantly influenced the purity, stability, yield, and composition of plant-derived exosomes, ultimately shaping their therapeutic potential [42].

## 6. Comparison between mammalian exosomes and PDELNs

Mammalian exosomes and PDELNs share similarities in their ability to mediate intercellular communication and deliver bioactive molecules [81]. However, they differ significantly in terms of therapeutic efficiency, stability, and scalability. Mammalian exosomes exhibit strong tissue targeting and endogenous biomolecule delivery capabilities, making them effective therapeutic agents. However, their production is costly and requires complex cell culture conditions [82]. In contrast, PDELNs are more abundant, cost-effective, and demonstrate good stability under physiological conditions, but their therapeutic efficiency in some contexts remains under investigation [15].

To provide a clearer comparison between PDELNs and mammalian exosomes, Table 2 summarizes their advantages and limitations.

**Table 2.** Comparison of advantages and disadvantages between PDELNs and mammalian exosomes.

Feature	PDELNs	Mammalian Exosomes
High yield	High yield, widely available, easy for large-scale production	Low yield, high production cost, dependent on cell culture
Stability	Stable under extreme pH conditions, gastrointestinal tract, and long-term storage	Requires low-temperature storage, stability influenced by the biological environment
Toxicity and immunogenicity	Low immunogenicity, orally administrable, good biocompatibility	May trigger immune responses, especially from heterologous sources
Therapeutic efficiency	Exhibits intrinsic bioactivity (e.g. anti-inflammatory, antioxidative), but targeted delivery needs optimization	Strong tissue-specific delivery capabilities, effective for precision medicine
Crossing BBB	Some PDELNs have shown the ability to cross the BBB via nasal-brain routes or active transport	Certain mammalian exosomes can penetrate the BBB, but efficiency varies by cell source
Scalability	Can be extracted from large quantities of plants, cost-effective, industrial-scale preparation possible	High production cost, complex cell culture, limited scalability
Heterogeneity	Composition varies significantly depending on the plant source	Composition varies depending on the cell source
Biomolecule composition	Rich in secondary metabolites, polysaccharides, small RNAs, flavonoids, etc.	Contains miRNAs, proteins (CD9/CD63/CD81), and other biomolecules

## 7. Therapeutic potential of PDELNs in CNS diseases

After exploring the multiple advantages of PDELNs and their biodistribution in the CNS, their potential applications in CNS diseases are particularly noteworthy. Owing to their stability, low immunogenicity, and ability to cross the BBB, PDELNs have significant therapeutic value for treating CNS disorders. This section focuses on the prospects and mechanisms of action of PDELNs in the treatment of CNS diseases (Table 3).

**Table 3.** Application of PDELNs in CNS diseases.

Name of disease	Plant source	In Vivo	In Vitro	Main findings	Animal dosing method	Ref.
Development of neural differentiation	Ginseng	Full-excisional wound site in rats	BMSCs	GExos have excellent efficiency by stimulating the neural differentiation of BMSCs by transferring the incorporated miRNAs to BMSCs efficiently	Cross-linked gel dressing containing GExos applied to wound sites	[55]
Parkinson’s disease	Carrot	No *in vivo* experiment conducted	human neuroblastoma SHSY5Y cells	Carex significantly inhibited reactive oxygen species generation and apoptosis in SH-SY5Y cells by regulating the antioxidative molecule expression. Carex decreased oxidative stress and increased cell viability in a 6-hydroxydopamine induced PD model.	N/A	[26]
	*Pueraria* *lobata*	PD mouse model (intranasal /intravenous) delivery of exosomes)	SH-SY5Y cells (neuroblastoma cells)	Pu-Exos enhance mitophagy via the PINK1-Parkin pathway, restore mitochondrial respiratory chain complexes I and V, and increase ATP production in SH-SY5Y cells. In PD mice, Pu-Exos-PR (engineered exosomes with DSPE-PEG-RVG) improved motor coordination, reduced neuronal degeneration, and enhanced survival of dopaminergic neuron survival.	Intranasal or intravenous delivery of exosomes	[83]
Ischemic Brain Injury	Momordica charantia	Middle cerebral artery occlusion (MCAO) in rats	HT22 mouse hippocampal neuronal cells	MC-ELNs protect against ischemic brain injury by inhibitingmatrix metalloproteinase-9 and activating the AKT/GSK3β signaling pathway, reducing neuronal apoptosis, protecting BBB integrity, and decreasing infarct size.	Intravenous injection of MC-ELNs	[84]
Alcohol-induced brain inflammation	Oat	Chronic alcohol diet in C57BL/6 mice	BV2 microglial cells	oat-ELNs inhibit alcohol-induced brain inflammation by blocking inflammatory cytokines (IL-6, IL-1β, TNF-α) through the Dectin-1/HPCA/Rab11a pathway. oat-ELNs prevented activation of the NF-κB signaling pathway and reduced neuronal apoptosis in the hippocampus, improving memory in alcohol-treated mice.	Oral administration of oat nanoparticles	[76]
Neuro- inflammation	*Allium tuberosum*	No *in vivo* experiment conducted	BV-2 microglial and MG-6 cells	A-ELNs significantly reduced LPS-induced nitric oxide, TNF-α,and IL-6 levels in BV-2 and MG-6 cells. A-ELNs also decreased the expression of inducible nitric oxide synthase and increased heme oxygenase-1 mRNA expression, showing strong anti-inflammatory effects. Dex-A-ELNs (dexamethasone-incorporated A-ELNs) enhanced anti-inflammatory properties.	N/A	[87]
Ischemia-reperfusion (I/R) injury	*Panax* *notoginseng*	tMCAO model in Sprague– Dawley rats	Primary microglia cells of neonatal SD rats	P-ELNs crossed the BBB, decreased infarct volume, improved motor function and altered microglial polarization from M1 to M2. The lipids in these nanoparticles were the key bioactive component.	Intravenous or intranasal delivery of nanoparticles	[85]
Hemorrhagic transformation (HT) in cerebral I/R injury	Momordica charantia	MCAO model in rats	Mouse brain microvascular endothelial cells (b.End3)	MC-ELNs reduced hemorrhagic transformation, improved neurological function, and protected BBB integrity in delayed t-PA treated I/R rats. MC-ELNs reduced ONOO − levels, inhibited the HMGB1/MMP-9 signaling pathway, decreased cell apoptosis, and prevented BBB disruption.	Intravenous administration of nanovesicles	[86]
Glioma	Ginseng	Orthotopic C6 glioma in Wistar rats, Balb/C mice	C6 glioma cells	GENs crossed the BBB, induced apoptosis in glioma cells, and modulated TEM by inhibiting M2 macrophage polarization and promoting M1 macrophage responses. GENs significantly reduced tumor volume and prolonged survival in glioma-bearing mice.	Intravenous delivery of nanoparticles	[14]

**Figure 2.**
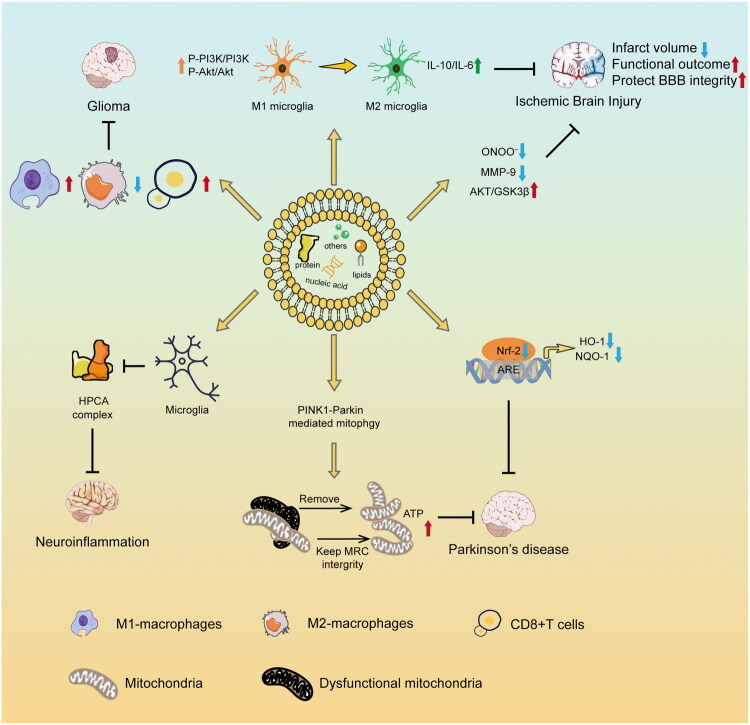
Potential therapeutic mechanisms of PDELNs in CNS diseases.

As illustrated in Figure 2, PDELNs have shown potential for treating neuroinflammation, ischemic brain injury, and PD by regulating immune cell function, promoting mitophagy, and activating antioxidant pathways. These mechanisms make PDELNs promising candidates for CNS disease therapy, as they may help alleviate neural inflammation, reduce oxidative stress, and support neuronal survival and recovery.

### 7.1. Therapeutic applications of PDELNs in CNS disorders

Xu et al. discovered that GExos can effectively promote the neural differentiation of BMSCs through miRNA delivery, demonstrating its advantages in tissue regeneration. However, this study was primarily based on *in vitro* experiments, with *in vivo* validation conducted only in a skin wound model. However, the therapeutic efficacy of GExos in CNS diseases has not yet been evaluated [55]. In contrast, GENs studied by Kim et al. showed outstanding effects in an animal model of glioblastoma. GENs cross the BBB and modulate the tumor microenvironment (TME), significantly inhibiting glioblastoma growth and prolonging survival in animal models [14]. This study confirmed the antitumor effects of GENs and validated their excellent biocompatibility and *in vivo* stability, showcasing their immense potential in CNS disease treatment.

Exosomes derived from *Pueraria lobata* (Pu-Exos) also showed notable neuroprotective effects in a PD animal model of PD. Pu-Exos, administered intranasally, successfully crossed the BBB and acted directly on the CNS. By enhancing mitophagy and restoring energy metabolism, Pu-Exos significantly improved the motor functions of PD model animals, validating their neuroprotective effects and therapeutic potential *in vivo* [83]. In addition, Carex demonstrated significant antioxidant and anti-apoptotic effects in *in vitro* experiments using PD models. However, their efficacy *in vivo* remains to be determined owing to the lack of *in vivo* studies.

Similarly, when treating ischemic brain injury, Momordica charantia-derived exosome-like nanoparticles (MC-ELNs) and *Panax notoginseng*-derived exosome-like nanoparticles (P-ELNs) exert neuroprotective effects through the AKT/GSK3β and microglial polarization pathways, respectively. *In vivo* studies have revealed that both can protect the BBB and reduce the volume of infarcts in the brain [84,85]. P-ELNs exhibit complex immune modulation functions by converting pro-inflammatory M1-type microglia into anti-inflammatory M2-type microglia, highlighting their significant advantages in managing CNS inflammation. In comparison, the primary mechanism of MC-ELNs is to protect the BBB and reduce apoptosis; however, their role in immune modulation still requires further investigation [84,86]. Although both showed promising neuroprotective and brain-damage-reducing effects, P-ELNs may have broader applications in inflammation-related CNS diseases because of their multiple mechanisms of action.

Finally, *Allium tuberosum*-derived exosome-like nanoparticles (A-ELNs) and oat-derived exosome-like nanoparticles (oat-ELNs) exert potent anti-inflammatory effects in the treatment of neuroinflammation and alcohol-induced brain inflammation. oat-ELNs, targeting microglial cells, regulate the assembly of the HPCA/Rab11a/Dectin-1 complex, inhibiting alcohol-induced brain inflammation, and improving cognitive function [76]. In contrast, A-ELNs exhibit significant anti-neuroinflammatory effects by reducing inflammatory factors and oxidative stress [87].

In conclusion, PDELNs demonstrate a significant potential for the treatment of CNS diseases. Different delivery routes offer distinct advantages and limitations, with key benefits including excellent biocompatibility, low immunogenicity, and efficient drug delivery capabilities. PDELNs from various sources have demonstrated multiple mechanisms in *in vitro* and *in vivo* experiments, including neuroprotection, restoration of mitochondrial function, immune modulation, and anti-inflammatory effects. These findings provide important evidence and insights for the future application of PDELNs in CNS disease therapy.

### 7.2. BBB penetration and delivery mechanisms

Multiple studies have demonstrated that PDELNs can cross the BBB. For instance, Peng et al. found that Pu-Exos effectively penetrated an *in vitro* BBB model and accumulated in mouse brain tissues after intravenous administration [83]. Similarly, Qi and Wang et al. developed MC-ELNs with diameters less than 200 nm. Upon intravenous injection into ischemic rats, fluorescence signals significantly increased in the striatal region, further confirming the ability of MC-ELNs to traverse the BBB and accumulate in ischemic brain regions [84,88].

Despite these promising findings, the precise transport mechanisms by which PDELNs cross the BBB remain unclear. To investigate these mechanisms, Wang et al. employed an *in vitro* BBB model, *in vivo* imaging, and 3D tumor spheroid models along with low-temperature treatment and various endocytosis inhibitors. Their findings indicated that GENs primarily enter brain tissues *via* phagocytosis, with paracellular transport playing a relatively minor role in the BBB crossing process [14]. However, Zhang et al. revealed that BBB penetration by oat-ELNs involves a more complex mechanism. *In vitro* studies confirmed that oat-ELNs can cross endothelial cell layers, and temperature-dependent experiments further suggested that their transport was mediated by both active transport and passive diffusion. *In vivo* tracing and quantitative analysis also demonstrated that orally administered oat-ELNs could penetrate the BBB, albeit at a relatively low efficiency. Nevertheless, considering the complexity of oral administration and the restrictive nature of the BBB, this study provides compelling evidence supporting the potential of oat-ELNs for brain delivery [76].

### 7.3. Targeting strategies and delivery routes

In addition to BBB penetration, the complexity of brain disorders presents an additional challenge. Different neurological diseases involve distinct receptor types and distributions, which directly affect the targeting efficiency of the delivery systems. Achieving precise delivery necessitated a comprehensive assessment of PDELNs’ binding affinity of PDELNs to disease-associated receptors and optimization of surface modifications to enhance targeting capabilities.

Surface modification of mammalian exosomes has been widely employed to improve brain-targeting efficiency and enhance drug delivery. For example, the genetic fusion of Lamp2b with a 29-amino-acid peptide fragment derived from RVG allowed exosomes to preferentially bind to nicotinic acetylcholine receptors. Studies have demonstrated that RVG-Lamp2b-modified EVs efficiently deliver siRNA to the mouse brain, leading to knockdown of BACE1, a gene implicated in AD progression [89]. Similarly, mesenchymal stem cell-derived RVG-modified EVs have been used to deliver therapeutic agents to alleviate neuroinflammation and improve neurological function in AD mouse models [90].

Similar approaches have also been explored for PDELNs. Zhuang et al. extracted GDELNs and modified them with FA ligands to deliver miR-17 to folate receptor-positive brain tumor cells. Intranasal administration of these modified nanovesicles results in rapid brain entry and preferential uptake by tumor cells, effectively inhibiting tumor growth [21]. Peng et al. engineered Pu-Exos by conjugating them with DPR, forming Pu-Exos-PR, which exhibited a 1.45-fold increase in brain accumulation compared with unmodified Pu-Exos [83]. Furthermore, Zhang et al. discovered that oat-ELNs, naturally rich in β-glucan, specifically bind to HPCA on microglial cells, enabling direct uptake into microglia. This finding suggests that certain PDELNs can achieve targeted delivery through their intrinsic bioactive components without additional surface modifications, providing a novel perspective on the application of PDELNs in neurological disorders [76].

In addition, delivery routes play a crucial role in determining the distribution and efficiency of PDELNs in the brain. Intravenous injection remains the most common route of administration, yet its effectiveness is often limited by the restrictive nature of the BBB. In contrast, intranasal administration has gained significant attention because it bypasses the BBB and enables direct drug delivery *via* the nose-to-brain pathway. Zhuang et al. demonstrated that intranasal administration of FA-modified PDELNs led to rapid brain entry and preferential tumor cell uptake, effectively suppressing tumor growth [21]. Peng et al. compared different delivery routes for Pu-Exos-PR and found that six hours post-administration, intravenously injected Pu-Exos-PR exhibited significantly higher brain accumulation. However, at the 12-hour mark, intranasally administered Pu-Exos-PR demonstrated superior brain distribution. This phenomenon was attributed to PEG chain modifications, which enhanced mucosal adhesion and promoted drug diffusion and mucus penetration [83].

Thus, optimizing PDELN-based delivery strategies requires not only effective targeting modifications, but also careful selection of administration routes based on drug properties and desired therapeutic outcomes to achieve efficient brain-targeted delivery.

## 8. Clinical trials of PDELNs

Clinical research on PDELNs is still in its early stages with only a few ongoing trials (Table 4). A Phase I trial (NCT01668849) evaluated grape-derived ELNs as an oral supplement for oral mucositis, focusing on pain reduction and immune biomarker assessment [91]. GExos and aloe-derived ELNs have been investigated for their potential role in improving insulin resistance and reducing chronic inflammation in patients with polycystic ovary syndrome (PCOS), although publicly available data remain limited. Curcumin-loaded PDELNs were investigated in an active but non-recruiting Phase I trial (NCT01294072) for colorectal cancer, with preliminary findings suggesting potential tumor suppression, although official results have yet to be published [92]. Further clinical trials adhering to Good Manufacturing Practice (GMP) guidelines are deemed essential to validate the therapeutic potential of PDELNs.

**Table 4.** Clinical trials of PDELNs.

Trial ID	Study Phase	PDELNs Source	Therapeutic Application	Study Insights	Current Status
NCT01668849	Phase I	Grape-derived ELNs	Oral mucositis (oral supplement)	Evaluating pain relief and immune biomarker response	Active, not recruiting
NCT03493984	Preliminary research	Ginger- and Aloe-derived ELNs	Insulin resistance and chronic inflammation (PCOS)	Limited publicly available data; aims to assess metabolic effects	Recruiting
NCT01294072	Phase I	Curcumin-loaded PDELNs	Colorectal cancer (oral delivery)	Investigating tumor suppression; results not yet published	Active, not recruiting

## 9. Ideal nanomedicine candidate for CNS

An ideal nanomedicine for CNS diseases must integrate key properties to effectively traverse the BBB and achieve targeted therapeutic outcomes. First, it is required to exhibit high BBB penetration efficiency while maintaining excellent biocompatibility and safety. Second, precise targeting capabilities and disease specificity are essential for enhancing therapeutic efficacy and minimizing off-target effects. Additionally, optimized drug-loading capacity and controlled release mechanisms are crucial for sustained treatment benefits. Finally, scalability and cost-effectiveness are fundamental for successful clinical translation, ensuring the feasibility of large-scale production and widespread application.

## 10. Summary and future perspectives

PDELNs are regarded as critical regulatory factors across species, serving not only as mediators of inter-species communication, but also as potential in treating human diseases. PDELNs have shown broad application prospects in recent years, particularly for the treatment of CNS diseases. As natural bioactive carriers, PDELNs offer new possibilities for CNS therapies owing to their low immunogenicity, high biocompatibility, and ability to cross the BBB.

This review focuses on the preparation and isolation techniques of PDELNs, analyzes their physicochemical properties, and elaborates on their potential applications in neurological diseases such as AD, PD, and ischemic brain injury. The components of PDELNs, including lipids, proteins, and RNA molecules, play critical roles in drug delivery and intercellular signaling. Compared with traditional synthetic nanoparticles and mammalian exosomes, PDELNs exhibit unique advantages. First, PDELNs originate from edible plants and exhibit excellent biocompatibility. They effectively avoide the potential immune rejection associated with synthetic nanoparticles and certain mammalian exosomes [93,94]. For example, G-ELNs demonstrate good tolerance in a mouse model, with no significant adverse effects [95]. Second, PDELNs possess an inherent ability to cross physiological barriers. Their nanoscale size and lipid bilayer structure allow for efficient BBB penetration without additional modifications [84,88]. In contrast, synthetic nanoparticles often require surface modifications with targeting ligands, such as transferrin or lactoferrin, to traverse the BBB [96,97]. PDELNs provide a simpler transport mechanism; although PDELNs show promising properties, several challenges remain. First, the mechanisms by which PDELNs cross the BBB are not fully understood. Second, there are a limited number of *in vivo* studies. Additionally, the properties and functions of PDELNs from different plant sources have yet to be fully defined. While the future holds great promise, achieving large-scale applications requires further experimental validation and technological optimization.

Future research should focus on standardized preparation and optimization techniques. Although various methods have been developed for PDELNs preparation and isolation, laboratories differ significantly in yield, purity, and consistency. A key priority is to establish standardized preparation and characterization protocols to ensure reproducibility and consistency in the properties of PDELNs from different sources. Furthermore, identification of universal and specific markers for plant exosomes remains an urgent challenge. Developing PDELN-specific biomarkers not only facilitates more precise isolation but also improves the understanding of their biological functions and interactions with target cells [98]. Recent studies suggested that TET8 had been identified as a marker in *Arabidopsis*-derived ELNs and was thought to be involved in stress responses and intercellular communication [58]. However, whether these markers are universally applicable to PDELNs from various plant sources remains unclear, and surface modifications, such as ligand conjugation and genetic engineering, enhance PDELNs’ targeting efficiency and therapeutic potential of PDELNs. For example, FA-conjugated GDELNs selectively targeted folate receptor-positive brain tumor cells, demonstrating improved drug delivery capabilities [21].

Another critical aspect was the exploration of the molecular mechanisms of PDELNs in central nervous system diseases. Studies have shown that some PDELNs exhibit strong BBB penetration capabilities, with different PDELNs potentially utilizing different transport mechanisms. Factors such as particle size, surface charge, modification methods, and targeting molecules influence BBB crossing efficiency. In addition to the intrinsic properties of exosomes, factors such as the complexity of the brain and the route of administration also play a crucial role in determining the ability of PDELNs to traverse the BBB. For example, Zhuang et al. demonstrated that intranasally administered PDELNs entered the brain more rapidly and were more efficiently taken up by tumor cells than intravenously administered ones, effectively inhibiting tumor growth [21]. The BBB primarily consists of brain microvascular endothelial cells, which restrict the entry of exogenous substances into the central nervous system through tight junctions [99]. Moreover, microglia, astrocytes, and oligodendrocytes are essential for maintaining BBB stability, regulating neuroinflammation, and mediating pathological processes [100–103]. Therefore, elucidating the interactions between PDELNs and BBB endothelial cells as well as the neural microenvironment is critical for optimizing their BBB-crossing efficiency. For instance, Zhang et al. found that oat-ELNs specifically bind to HPCA on microglia, facilitating the direct uptake of PDELNs by these cells [76]. However, research on the molecular mechanisms underlying the ability of PDELNs to cross the BBB and target the neural microenvironment is limited. Future studies should leverage advanced molecular biology techniques to elucidate the role of PDELNs in treating neurological diseases.

Beyond traditional drug delivery, interdisciplinary research integrating nanomedicine, molecular biology, and artificial intelligence (AI) has further unlocked the potential of PDELNs. For instance, combining PDELNs with CRISPR gene editing technology facilitates precise gene therapy [104]. AI-assisted drug design optimized PDELNs’ formulation for personalized medicine, enhancing therapeutic efficacy [105].

Despite these promising prospects, transitioning PDELNs from preclinical research to clinical application presents several challenges. One major obstacle is the scalability of the PDELNs production. Current extraction and purification methods require optimization for large-scale manufacturing while maintaining consistency, yield, and purity. Another major obstacle is the long-term safety of PDELNs, as there is a lack of sufficient clinical trials to assess their potential risks, immune responses, and long-term effects in humans. Additionally, the cost-effectiveness of PDELNs production was evaluated in comparison with synthetic or mammalian-derived alternatives. Considering the environmental impact, PDELNs offer a more sustainable option owing to their plant origin and renewable nature, making them a potentially eco-friendly choice in nanomedicine.

## 11. Conclusion

PDELNs offer a novel solution for treating CNS diseases with a significant potential for application. Future research should address challenges such as unclear mechanisms and difficulties in large-scale production while validating their long-term safety and efficacy in *in vivo* studies. Through multidisciplinary collaboration and technological innovation, PDELNs are expected to provide new therapeutic options for patients with CNS diseases in the near future, and play a broader role in drug delivery and other fields.

## Data Availability

Data sharing is not applicable to this article as no new data were created or analyzed in this study.

## References

[1] Zhou X, Smith QR, Liu X. Brain penetrating peptides and peptide-drug conjugates to overcome the blood-brain barrier and target cns diseases. Wiley Interdiscip Rev Nanomed Nanobiotechnol. 2021;13(4):e1695. doi: 10.1002/wnan.1695. 33470550

[2] Piguet F, de Saint DT, Audouard E, et al. The challenge of gene therapy for neurological diseases: strategies and tools to achieve efficient delivery to the central nervous system. Hum Gene Ther. 2021;32(7–8):349–374. doi: 10.1089/hum.2020.105. 33167739

[3] Akhtar A, Andleeb A, Waris TS, et al. Neurodegenerative diseases and effective drug delivery: a review of challenges and novel therapeutics. J Control Release. 2021;330:1152–1167. doi: 10.1016/j.jconrel.2020.11.021. 33197487

[4] Cavaco M, Gaspar D, Arb CM, et al. Antibodies for the treatment of brain metastases, a dream or a reality? Pharmaceutics. 2020;12(1):62. doi: 10.3390/pharmaceutics12010062. 31940974 PMC7023012

[5] Wang D, Wang C, Wang L, et al. A comprehensive review in improving delivery of small-molecule chemotherapeutic agents overcoming the blood-brain/brain tumor barriers for glioblastoma treatment. Drug Deliv. 2019;26(1):551–565. doi: 10.1080/10717544.2019.1616235. 31928355 PMC6534214

[6] Padmakumar S, D’Souza A, Parayath NN, et al. Nucleic acid therapies for cns diseases: pathophysiology, targets, barriers, and delivery strategies. J Control Release. 2022;352:121–145. doi: 10.1016/j.jconrel.2022.10.018. 36252748

[7] Kalluri R, Lebleu VS. The biology, function, and biomedical applications of exosomes. Science. 2020;367(6478):eaau6977. doi: 10.1126/science.aau6977. PMC7717626 32029601

[8] Fan Y, Chen Z, Zhang M. Role of exosomes in the pathogenesis, diagnosis, and treatment of central nervous system diseases. J Transl Med. 2022;20(1):291. doi: 10.1186/s12967-022-03493-6. 35761337 PMC9235237

[9] Halperin W, Jensen WA. Ultrastructural changes during growth and embryogenesis in carrot cell cultures. J Ultrastruct Res. 1967;18(3):428–443. doi: 10.1016/s0022-5320(67)80128-x. 6025110

[10] Regente M, Corti-Monzon G, Maldonado AM, et al. Vesicular fractions of sunflower apoplastic fluids are associated with potential exosome marker proteins. FEBS Lett. 2009;583(20):3363–3366. doi: 10.1016/j.febslet.2009.09.041. 19796642

[11] Kim J, Li S, Zhang S, et al. Plant-derived exosome-like nanoparticles and their therapeutic activities. Asian J Pharm Sci. 2022;17(1):53–69. doi: 10.1016/j.ajps.2021.05.006. 35261644 PMC8888139

[12] Ju S, Mu J, Dokland T, et al. Grape exosome-like nanoparticles induce intestinal stem cells and protect mice from dss-induced colitis. Mol Ther. 2013;21(7):1345–1357. doi: 10.1038/mt.2013.64. 23752315 PMC3702113

[13] Feng J, Xiu Q, Huang Y, et al. Plant-derived vesicle-like nanoparticles as promising biotherapeutic tools: present and future. Adv Mater. 2023;35(24):e2207826. doi: 10.1002/adma.202207826. 36592157

[14] Kim J, Zhu Y, Chen S, et al. Anti-glioma effect of ginseng-derived exosomes-like nanoparticles by active blood-brain-barrier penetration and tumor microenvironment modulation. J Nanobiotechnology. 2023;21(1):253. doi: 10.1186/s12951-023-02006-x. 37542285 PMC10401762

[15] Yi Q, Xu Z, Thakur A, et al. Current understanding of plant-derived exosome-like nanoparticles in regulating the inflammatory response and immune system microenvironment. Pharmacol Res. 2023;190:106733. doi: 10.1016/j.phrs.2023.106733. 36931541

[16] Bai C, Liu J, Zhang X, et al. Research status and challenges of plant-derived exosome-like nanoparticles, Biomed. Biomed Pharmacother. 2024;174:116543. doi: 10.1016/j.biopha.2024.116543. 38608523

[17] Thery C, Witwer KW, Aikawa E, et al. Minimal information for studies of extracellular vesicles 2018 (misev2018): a position statement of the international society for extracellular vesicles and update of the misev2014 guidelines. J. Extracell. Vesicles. 2018;7(1):1535750. 30637094 10.1080/20013078.2018.1535750 PMC6322352

[18] Chukhchin DG, Bolotova K, Sinelnikov I, et al. Exosomes in the phloem and xylem of woody plants. Planta. 2019;251(1):12. doi: 10.1007/s00425-019-03315-y. 31776666

[19] Liu Y, Wu S, Koo Y, et al. Characterization of and isolation methods for plant leaf nanovesicles and small extracellular vesicles, E.C. Tang, E.W. Chehab, N. de Val, J. Braam, A.K. Sood, editors. Nanomedicine 29; 2020, 102271. 10.1016/j.nano.2020.102271 32702466

[20] Huang Y, Wang S, Cai Q, et al. Effective methods for isolation and purification of extracellular vesicles from plants. J Integr Plant Biol. 2021;63(12):2020–2030. doi: 10.1111/jipb.13181. 34668639 PMC8972076

[21] Zhuang X, Teng Y, Samykutty A, et al. Grapefruit-derived nanovectors delivering therapeutic mir17 through an intranasal route inhibit brain tumor progression. Mol Ther. 2016;24(1):96–105. doi: 10.1038/mt.2015.188. 26444082 PMC4754550

[22] Seo K, Yoo JH, Kim J, et al. Ginseng-derived exosome-like nanovesicles extracted by sucrose gradient ultracentrifugation to inhibit osteoclast differentiation. Nanoscale. 2023;15(12):5798–5808. doi: 10.1039/d2nr07018a. 36857681

[23] Rutter BD, Innes RW. Extracellular vesicles isolated from the leaf apoplast carry stress-response proteins. Plant Physiol. 2017;173(1):728–741. doi: 10.1104/pp.16.01253. 27837092 PMC5210723

[24] Edelstein C, Pfaffinger D, Scanu AM. Advantages and limitations of density gradient ultracentrifugation in the fractionation of human serum lipoproteins: role of salts and sucrose. J Lipid Res. 1984;25(6):630–637. doi: 10.1016/S0022-2275(20)37776-2. 6747466

[25] Kang SJ, Kim SE, Seo M, et al. Suppression of inflammatory responses in macrophages by onion-derived extracellular vesicles. J. Ind. Eng. Chem. 2022;115:287–297. doi: 10.1016/j.jiec.2022.08.011.

[26] Kim DK, Rhee WJ. Antioxidative effects of carrot-derived nanovesicles in cardiomyoblast and neuroblastoma cells. Pharmaceutics. 2021;13(8):1203. doi: 10.3390/pharmaceutics13081203. 34452164 PMC8400689

[27] You JY, Kang SJ, Rhee WJ. Isolation of cabbage exosome-like nanovesicles and investigation of their biological activities in human cells. Bioact. Mater. 2021;6(12):4321–4332. doi: 10.1016/j.bioactmat.2021.04.023. 33997509 PMC8105599

[28] Berger E, Colosetti P, Jalabert A, et al. Use of nanovesicles from orange juice to reverse diet-induced gut modifications in diet-induced obese mice. Mol Ther Methods Clin Dev. 2020;18:880–892. doi: 10.1016/j.omtm.2020.08.009. 32953937 PMC7481887

[29] Hung JC, Hambleton G, Super M. Evaluation of two commercial jet nebulisers and three compressors for the nebulisation of antibiotics. Arch Dis Child. 1994;71(4):335–338. doi: 10.1136/adc.71.4.335. 7979528 PMC1030014

[30] Kalarikkal SP, Prasad D, Kasiappan R, et al. A cost-effective polyethylene glycol-based method for the isolation of functional edible nanoparticles from ginger rhizomes. Sci. Rep. 2020;10(1):4456. doi: 10.1038/s41598-020-61358-8. 32157137 PMC7064537

[31] Nguyen VQ, Um W, An JY, et al. Precipitation-mediated pegylation of plant-derived nanovesicles. Macromol Res. 2022;30(2):85–89. doi: 10.1007/s13233-022-0016-x.

[32] Suga T, Hirano M, Takayanagi M, et al. Restricted photorelease of biologically active molecules near the plasma membrane. Biochem Biophys Res Commun. 1998;253(2):423–430. doi: 10.1006/bbrc.1998.9153. 9878552

[33] Lange H, Gagliardi D. Catalytic activities, molecular connections, and biological functions of plant rna exosome complexes. Plant Cell. 2022;34(3):967–988. doi: 10.1093/plcell/koab310. 34954803 PMC8894942

[34] Garaeva L, Kamyshinsky R, Kil Y, et al. Delivery of functional exogenous proteins by plant-derived vesicles to human cells *in vitro*. Sci Rep. 2021;11(1):6489. doi: 10.1038/s41598-021-85833-y. 33753795 PMC7985202

[35] Sharma S, Mahanty M, Rahaman SG, et al. Avocado-derived extracellular vesicles loaded with ginkgetin and berberine prevent inflammation and macrophage foam cell formation. J Cell Mol Med. 2024;28(7):e18177. doi: 10.1111/jcmm.18177. 38494843 PMC10945093

[36] Bhattacharjee S. Dls and zeta potential - what they are and what they are not? J Control Release. 2016;235:337–351. doi: 10.1016/j.jconrel.2016.06.017. 27297779

[37] Chaturvedi S, Maheshwari D, Chawathe A, et al. Current analytical approaches for characterizing nanoparticle sizes in pharmaceutical research. J Nanopart Res. 2024;26(1):19. doi: 10.1007/s11051-023-05924-x.

[38] Sakai-Kato K, Yoshida K, Izutsu KI. Effect of surface charge on the size-dependent cellular internalization of liposomes. Chem Phys Lipids. 2019;224:104726. doi: 10.1016/j.chemphyslip.2019.01.004. 30660745

[39] Dobrovolskaia MA, Aggarwal P, Hall JB, et al. Preclinical studies to understand nanoparticle interaction with the immune system and its potential effects on nanoparticle biodistribution. Mol Pharm. 2008;5(4):487–495. doi: 10.1021/mp800032f. 18510338 PMC2613572

[40] Zhao Z, Lacombe J, Simon L, et al. Physical, biochemical, and biological characterization of olive-derived lipid nanovesicles for drug delivery applications. J Nanobiotechnology. 2024;22(1):720. doi: 10.1186/s12951-024-02964-w. 39558361 PMC11575425

[41] Bommakanti V, Banerjee M, Shah D, et al. An overview of synthesis, characterization, applications and associated adverse effects of bioactive nanoparticles. Environ Res. 2022;214(Pt 2):113919. doi: 10.1016/j.envres.2022.113919. 35863448

[42] Jang J, Jeong H, Jang E, et al. Isolation of high-purity and high-stability exosomes from ginseng. Front Plant Sci. 2022;13:1064412. doi: 10.3389/fpls.2022.1064412. 36714697 PMC9878552

[43] Jiyah A, Muhammad SA, Ibrahim A, et al. Plant-derived extracellular vesicles as potential smart nano drug delivery systems for antioxidant vitamins c and e in alzheimer’s disease. J. Drug Deliv. Sci. Technol. 2024;95:105618. doi: 10.1016/j.jddst.2024.105618

[44] Woith E, Melzig MF. Extracellular vesicles from fresh and dried plants-simultaneous purification and visualization using gel electrophoresis. Int J Mol Sci. 2019;20(2):357. doi: 10.3390/ijms20020357. 30654488 PMC6359398

[45] Lamparski HG, Metha-Damani A, Yao JY, et al. Production and characterization of clinical grade exosomes derived from dendritic cells. J Immunol Methods. 2002;270(2):211–226. doi: 10.1016/s0022-1759(02)00330-7. 12379326

[46] Andriolo G, Provasi E, Lo CV, et al. Exosomes from human cardiac progenitor cells for therapeutic applications: development of a gmp-grade manufacturing method. Front Physiol. 2018;9:1169. doi: 10.3389/fphys.2018.01169. 30197601 PMC6117231

[47] Pachler K, Lener T, Streif D, et al. A good manufacturing practice-grade standard protocol for exclusively ­human mesenchymal stromal cell-derived extracellular vesicles. Cytotherapy. 2017;19(4):458–472. doi: 10.1016/j.jcyt.2017.01.001. 28188071

[48] Samoil V, Dagenais M, Ganapathy V, et al. Vesicle-based secretion in schistosomes: analysis of protein and microrna (mirna) content of exosome-like vesicles derived from schistosoma mansoni. Sci Rep. 2018;8(1):3286. doi: 10.1038/s41598-018-21587-4. 29459722 PMC5818524

[49] Smith ZJ, Lee C, Rojalin T, et al. Single exosome study reveals subpopulations distributed among cell lines with variability related to membrane content. J. Extracell. Vesicles. 2015;4:28533. 26649679 10.3402/jev.v4.28533 PMC4673914

[50] Ren J, He W, Zheng L, et al. From structures to functions: insights into exosomes as promising drug delivery vehicles. Biomater Sci. 2016;4(6):910–921. doi: 10.1039/c5bm00583c. 26977477

[51] Wang X, Devaiah SP, Zhang W, et al. Signaling functions of phosphatidic acid. Prog Lipid Res. 2006;45(3):250–278. doi: 10.1016/j.plipres.2006.01.005. 16574237

[52] Dad HA, Gu T, Zhu A, et al. Plant exosome-like nanovesicles: emerging therapeutics and drug delivery nanoplatforms. Mol Ther. 2021;29(1):13–31. doi: 10.1016/j.ymthe.2020.11.030. 33278566 PMC7791080

[53] Pocsfalvi G, Turiák L, Ambrosone A, et al. Protein biocargo of citrus fruit-derived vesicles reveals heterogeneous transport and extracellular vesicle populations. J Plant Physiol. 2018;229:111–121. doi: 10.1016/j.jplph.2018.07.006. 30056374

[54] Wang Q, Zhuang X, Mu J, et al. Delivery of therapeutic agents by nanoparticles made of grapefruit-derived lipids. Nat Commun. 2013;4(1):1867. doi: 10.1038/ncomms2886. 23695661 PMC4396627

[55] Xu XH, Yuan TJ, Dad HA, et al. Plant exosomes as novel nanoplatforms for microrna transfer stimulate neural differentiation of stem cells *in vitro* and in vivo. Nano Lett. 2021;21(19):8151–8159. doi: 10.1021/acs.nanolett.1c02530. 34586821

[56] Stanly C, Moubarak M, Fiume I, et al. Membrane transporters in citrus clementina fruit juice-derived nanovesicles. Int J Mol Sci. 2019;20(24):6205. doi: 10.3390/ijms20246205. 31835328 PMC6941005

[57] Liang Y, Lehrich BM, Zheng S, et al. Emerging methods in biomarker identification for extracellular vesicle-based liquid biopsy. J Extracell Vesicles. 2021;10(7):e12090. doi: 10.1002/jev2.12090. 34012517 PMC8114032

[58] Cai Q, Qiao L, Wang M, et al. Plants send small rnas in extracellular vesicles to fungal pathogen to silence virulence genes. Science. 2018;360(6393):1126–1129. doi: 10.1126/science.aar4142. 29773668 PMC6442475

[59] Xu T, Zhu Y, Lin Z, et al. Evidence of cross-kingdom gene regulation by plant micrornas and possible reasons for inconsistencies. J Agric Food Chem. 2024;72(9):4564–4573. doi: 10.1021/acs.jafc.3c09097. 38391237

[60] Xiao J, Feng S, Wang X, et al. Identification of exosome-like nanoparticle-derived micrornas from 11 edible fruits and vegetables. PeerJ. 2018;6:e5186. doi: 10.7717/peerj.5186. 30083436 PMC6074755

[61] Zieve GW, Sauterer RA, Feeney RJ. Newly synthesized small nuclear rnas appear transiently in the cytoplasm. J Mol Biol. 1988;199(2):259–267. doi: 10.1016/0022-2836(88)90312-9. 3351925

[62] Baldrich P, Rutter BD, Karimi HZ, et al. Plant extracellular vesicles contain diverse small rna species and are enriched in 10- to 17-nucleotide "tiny" rnas. Plant Cell. 2019;31(2):315–324. doi: 10.1105/tpc.18.00872. 30705133 PMC6447009

[63] Mach J. Tyrna bubbles: extracellular vesicles carry 10-15-nucleotide small rnas and specific groups of micrornas. Plant Cell. 2019;31(3):558–558. doi: 10.1105/tpc.19.00130. 30814256 PMC6482626

[64] Mu J, Zhuang X, Wang Q, et al. Interspecies communication between plant and mouse gut host cells through edible plant derived exosome-like nanoparticles. Mol Nutr Food Res. 2014;58(7):1561–1573. doi: 10.1002/mnfr.201300729. 24842810 PMC4851829

[65] Lei C, Teng Y, He L, et al. Lemon exosome-like nanoparticles enhance stress survival of gut bacteria by rnase p-mediated specific trna decay. Iscience. 2021;24(6):102511. doi: 10.1016/j.isci.2021.102511. 34142028 PMC8188359

[66] Ou X, Wang H, Tie H, et al. Novel plant-derived exosome-like nanovesicles from catharanthus roseus: preparation, characterization, and immunostimulatory effect via tnf-alpha/nf-kappab/pu.1 axis. J Nanobiotechnol. 2023;21(1):160. doi: 10.1186/s12951-023-01919-x. PMC10199296 37210530

[67] Hwang JH, Park YS, Kim HS, et al. Yam-derived exosome-like nanovesicles stimulate osteoblast formation and prevent osteoporosis in mice. J Control Release. 2023;355:184–198. doi: 10.1016/j.jconrel.2023.01.071. 36736431

[68] Zhu MZ, Xu HM, Liang YJ, et al. Edible exosome-like nanoparticles from portulaca oleracea l mitigate dss-induced colitis via facilitating double-positive cd4(+)cd8(+)t cells expansion. J Nanobiotechnol. 2023;21(1):309. doi: 10.1186/s12951-023-02065-0. PMC10469825 37653406

[69] Umezu T, Takanashi M, Murakami Y, et al. Acerola exosome-like nanovesicles to systemically deliver nucleic acid medicine via oral administration. Mol Ther Methods Clin Dev. 2021;21:199–208. doi: 10.1016/j.omtm.2021.03.006. 33850951 PMC8010214

[70] Cong M, Tan S, Li S, et al. Technology insight: plant-derived vesicles-how far from the clinical biotherapeutics and therapeutic drug carriers? Adv Drug Deliv Rev. 2022;182:114108. doi: 10.1016/j.addr.2021.114108. 34990792

[71] Chen T, Ma B, Lu S, et al. Cucumber-derived nanovesicles containing cucurbitacin b for non-small cell lung cancer therapy. Int J Nanomed. 2022;17:3583–3599. doi: 10.2147/IJN.S362244. PMC9376005 35974872

[72] Stanly C, Alfieri M, Ambrosone A, et al. Grapefruit-derived micro and nanovesicles show distinct metabolome profiles and anticancer activities in the a375 human melanoma cell line. Cells. 2020;9(12):2722. doi: 10.3390/cells9122722. 33371199 PMC7766354

[73] Karamanidou T, Tsouknidas A. Plant-derived extracellular vesicles as therapeutic nanocarriers. Int J Mol Sci. 2021;23(1):191. doi: 10.3390/ijms23010191. 35008617 PMC8745116

[74] Cao M, Yan H, Han X, et al. Ginseng-derived nanoparticles alter macrophage polarization to inhibit melanoma growth. J Immunother Cancer. 2019;7(1):326. doi: 10.1186/s40425-019-0817-4. 31775862 PMC6882204

[75] Han X, Wei Q, Lv Y, et al. Ginseng-derived nanoparticles potentiate immune checkpoint antibody efficacy by reprogramming the cold tumor microenvironment. Mol Ther. 2022;30(1):327–340. doi: 10.1016/j.ymthe.2021.08.028. 34450250 PMC8753455

[76] Xu F, Mu J, Teng Y, et al. Restoring oat nanoparticles mediated brain memory function of mice fed alcohol by sorting inflammatory dectin-1 complex into microglial exosomes. Small. 2022;18(6):e2105385. doi: 10.1002/smll.202105385. 34897972 PMC8858573

[77] Wang Q, Ren Y, Mu J, et al. Grapefruit-derived nanovectors use an activated leukocyte trafficking pathway to deliver therapeutic agents to inflammatory tumor sites. Cancer Res. 2015;75(12):2520–2529. doi: 10.1158/0008-5472.CAN-14-3095. 25883092 PMC4470740

[78] Wang Y, Wei Y, Liao H, et al. Plant exosome-like nanoparticles as biological shuttles for transdermal drug delivery. Bioeng Basel. 2023;10(1):104. doi: 10.3390/bioengineering10010104. PMC9854743 36671676

[79] Zheng M, Chavda VP, Vaghela DA, et al. Plant-derived exosomes in therapeutic nanomedicine, paving the path toward precision medicine. Phytomedicine. 2024;135:156087. doi: 10.1016/j.phymed.2024.156087. 39388922

[80] Zhao B, Lin H, Jiang X, et al. Exosome-like nanoparticles derived from fruits, vegetables, and herbs: innovative strategies of therapeutic and drug delivery. Theranostics. 2024;14(12):4598–4621. doi: 10.7150/thno.97096. 39239509 PMC11373634

[81] Sall IM, Flaviu TA. Plant and mammalian-derived extracellular vesicles: a new therapeutic approach for the future. Front Bioeng Biotechnol. 2023;11:1215650. doi: 10.3389/fbioe.2023.1215650. 37781539 PMC10534050

[82] Richter M, Vader P, Fuhrmann G. Approaches to surface engineering of extracellular vesicles. Adv Drug Deliv Rev. 2021;173:416–426. doi: 10.1016/j.addr.2021.03.020. 33831479

[83] Xu Y, Yan G, Zhao J, et al. Plant-derived exosomes as cell homogeneous nanoplatforms for brain biomacromolecules delivery ameliorate mitochondrial dysfunction against parkinson’s disease. Nano Today. 2024;58:102438. doi: 10.1016/j.nantod.2024.102438.

[84] Cai H, Huang LY, Hong R, et al. Momordica charantia exosome-like nanoparticles exert neuroprotective effects against ischemic brain injury via inhibiting matrix metalloproteinase 9 and activating the akt/gsk3beta signaling pathway. Front Pharmacol. 2022;13:908830. doi: 10.3389/fphar.2022.908830. 35814200 PMC9263912

[85] Li S, Zhang R, Wang A, et al. Panax notoginseng: derived exosome-like nanoparticles attenuate ischemia reperfusion injury via altering microglia polarization. J Nanobiotechnoly. 2023;21(1):416. doi: 10.1186/s12951-023-02161-1. PMC10636993 37946257

[86] Ye C, Yan C, Bian SJ, et al. Momordica charantia l.-Derived exosome-like nanovesicles stabilize p62 expression to ameliorate doxorubicin cardiotoxicity. J Nanobiotechnol. 2024;22(1):464. doi: 10.1186/s12951-024-02705-z. PMC11297753 39095755

[87] Ishida T, Kawada K, Jobu K, et al. Exosome-like nanoparticles derived from allium tuberosum prevent neuroinflammation in microglia-like cells. J Pharm Pharmacol. 2023;75(10):1322–1331. doi: 10.1093/jpp/rgad062. 37390476

[88] Wang W, Wang P, Liang Z, et al. Exosome-like nanovesicles derived from momordica charantia ameliorate delayed t-pa thrombolysis-induced hemorrhagic transformation by inhibiting the onoo−/hmgb1/mmp-9 pathway. J Funct Foods. 2024;114:106086. doi: 10.1016/j.jff.2024.106086.

[89] Alvarez-Erviti L, Seow Y, Yin H, et al. Delivery of sirna to the mouse brain by systemic injection of targeted exosomes. Nat Biotechnol. 2011;29(4):341–345. doi: 10.1038/nbt.1807. 21423189

[90] Cui GH, Guo HD, Li H, et al. Rvg-modified exosomes derived from mesenchymal stem cells rescue memory deficits by regulating inflammatory responses in a mouse model of alzheimer’s disease. Immun Ageing. 2019;16:10. doi: 10.1186/s12979-019-0150-2. 31114624 PMC6515654

[91] Redman R. Edible plant exosome ability to prevent oral mucositis associated with chemoradiation treatment of head and neck cancer. 2012.

[92] Miller D. Study investigating the ability of plant exosomes to deliver curcumin to normal and colon cancer tissue. 2011.

[93] Naz S, Gul A, Zia M. Toxicity of copper oxide nanoparticles: a review study. IET Nanobiotechnol. 2020;14(1):1–13. doi: 10.1049/iet-nbt.2019.0176. 31935671 PMC8676634

[94] Ha DH, Kim SD, Lee J, et al. Toxicological evaluation of exosomes derived from human adipose tissue-derived mesenchymal stem/stromal cells. Regul Toxicol Pharmacol. 2020;115:104686. doi: 10.1016/j.yrtph.2020.104686. 32450131

[95] Zhuang X, Deng ZB, Mu J, et al. Ginger-derived nanoparticles protect against alcohol-induced liver damage. J Extracell Vesicles. 2015;4:28713. doi: 10.3402/jev.v4.28713. 26610593 PMC4662062

[96] Johnsen KB, Burkhart A, Thomsen LB, et al. Targeting the transferrin receptor for brain drug delivery. Prog Neurobiol. 2019;181:101665. doi: 10.1016/j.pneurobio.2019.101665. 31376426

[97] Janjua TI, Cao Y, Ahmed-Cox A, et al. Efficient delivery of temozolomide using ultrasmall large-pore silica nanoparticles for glioblastoma. J Control Release. 2023;357:161–174. doi: 10.1016/j.jconrel.2023.03.040. 36965857

[98] Lai JJ, Chau ZL, Chen SY, et al. Exosome processing and characterization approaches for research and technology development. Adv Sci (Weinh). 2022;9(15):e2103222. doi: 10.1002/advs.202103222. 35332686 PMC9130923

[99] Palma-Florez S, Lopez-Canosa A, Moralez-Zavala F, et al. Bbb-on-a-chip with integrated micro-teer for permeability evaluation of multi-functionalized gold nanorods against alzheimer’s disease. J Nanobiotechnol. 2023;21(1):115. doi: 10.1186/s12951-023-01798-2. PMC10053726 36978078

[100] Battaglia S, Nazzi C, Lonsdorf TB, et al. Neuropsychobiology of fear-induced bradycardia in humans: progress and pitfalls. Mol Psychiatry. 2024;29(12):3826–3840. doi: 10.1038/s41380-024-02600-x. 38862673 PMC11609102

[101] Nazzi C, Avenanti A, Battaglia S. The involvement of antioxidants in cognitive decline and neurodegeneration: mens sana in corpore sano. Antioxidants. 2024;13(6):701. doi: 10.3390/antiox13060701. 38929140 PMC11200558

[102] Battaglia S, Avenanti A, Vécsei L, et al. Neural correlates and molecular mechanisms of memory and learning. Int J Mol Sci. 2024;25(5):2724. doi: 10.3390/ijms25052724. 38473973 PMC10931689

[103] Gregorio FD, Battaglia S. The intricate brain-body interaction in psychiatric and neurological diseases. Adv Clin Exp Med. 2024;33(4):321–326. doi: 10.17219/acem/185689. 38515256

[104] Hillman T. The use of plant-derived exosome-like nanoparticles as a delivery system of crispr/cas9-based therapeutics for editing long non-coding rnas in cancer colon cells. Front Oncol. 2023;13:1194350. doi: 10.3389/fonc.2023.1194350. 37388221 PMC10301836

[105] Cao M, Diao N, Cai X, et al. Plant exosome nanovesicles (pens): green delivery platforms. Mater Horiz. 2023;10(10):3879–3894. doi: 10.1039/d3mh01030a. 37671650

